# Gastrointestinal Hemorrhage in Warfarin Anticoagulated Patients: Incidence, Risk Factor, Management, and Outcome

**DOI:** 10.1155/2014/463767

**Published:** 2014-05-29

**Authors:** Wen-Chi Chen, Yan-Hua Chen, Ping-I Hsu, Feng-Woei Tsay, Hoi-Hung Chan, Jin-Shiung Cheng, Kwok-Hung Lai

**Affiliations:** ^1^Division of Gastroenterology, Department of Medicine, Kaohsiung Veterans General Hospital, 386 Ta-Chung 1st Road, Kaohsiung 81362, Taiwan; ^2^Faculty of Medicine, School of Medicine, National Yang-Ming University, Taipei 11221, Taiwan; ^3^Department of Chemistry, College of Science, National Kaohsiung Normal University, Kaohsiung 82444, Taiwan; ^4^Department of Sport, Health & Leisure, Cheng Shiu University, Kaohsiung 83347, Taiwan

## Abstract

*Background.* Warfarin reduces the incidence of thromboembolism but increases the risk of gastrointestinal bleeding (GIB). GIB during warfarin anticoagulation is rarely evaluated in Asian patients. *Aims.* This study aimed at investigating the incidence, risk factors, management, and outcome of GIB in Taiwanese patients treated with warfarin. *Methods.* We analyzed a cohort of warfarin anticoagulated patients between July 1993 and May 2012. Clinical data were retrieved in a chart-reviewing manner. *Results.* A total of 401 warfarin anticoagulated patients were enrolled. The incidence of GIB was 3.9% per patient-years. Multivariate analysis with Cox regression showed that age >65 years old (RR: 2.5, 95% CI: 1.2–5.5), a mean international normalized ratio >2.1 (RR: 2.1, 95% CI: 1.0–4.2), a history of GIB (RR: 5.1, 95% CI: 1.9–13.5), and cirrhosis (RR: 6.9, 95% CI: 2.0–24.5) were independent factors predicting GIB. 27.3% of the GIB patients had rebleeding after restarting warfarin while thromboembolic events were found in 16.7% of the patients discontinuing warfarin therapy. *Conclusions.* Warfarin was associated with a significant incidence of GIB in Taiwanese patients. The intensity of anticoagulation should be monitored closely during warfarin therapy, especially in patients with risk factors of GIB.

## 1. Introduction

Warfarin is currently the most commonly used oral anticoagulant worldwide. It produces an anticoagulant effect by interfering with the cyclic interconversion of vitamin K and its 2,3-epoxide (vitamin K epoxide) [1]. The indications of warfarin include prevention of venous thromboembolism, prevention of systemic thromboembolism and stroke in patients with prosthetic heart valves and atrial fibrillation, primary prevention of myocardial infarction, and prevention of stroke, recurrent infarction, and death in the management of acute myocardial infarction [2]. However, warfarin has a narrow therapeutic window, wide variability in dose-response across individuals, and a significant number of drug and dietary interactions and requires close laboratory monitoring with frequent dose adjustment [1, 2].

Gastrointestinal bleeding (GIB) is one of the severe bleeding complications of warfarin anticoagulation and occurs in up to 12% of cases [3]. Several factors that influence the source and severity of GIB in patients taking warfarin are identified including prolonged prothrombin time, concomitant use of aspirin, advanced age, previous GIB, atrial fibrillation, and coexisting conditions such as renal insufficiency and anemia [4]. However, the correlation between some of these factors, for instance, advanced age, and GIB is controversial [5].

New oral anticoagulant agents are direct and selective inhibitors of a specific step or enzyme of the coagulation cascade. They have been shown to be effective in the prevention and treatment of various thromboembolic diseases with more predictable anticoagulant response and no need for close laboratory monitoring. However, new oral anticoagulants still have some limitations. Drug-drug interactions, difficulty in monitoring the anticoagulant effect in patients with severe renal and liver failure, the much more expensive prices compared with warfarin, and, most importantly, lack of a specific antidote are the major drawbacks of these agents [6]. A recent meta-analysis reveals that new generation of oral anticoagulants results in a significantly higher risk of GIB compared with warfarin [7]. Besides, new oral anticoagulants are not cost-effective when compared with warfarin in patients with atrial fibrillation [8]. Taking these together, warfarin remains a widely used anticoagulant before more promising agents are available. As a result, a more detailed understanding of the use of warfarin and its bleeding complication is necessary while managing the patients.

Average warfarin dose required to maintain the international normalized ratio (INR) between 2.0 and 3.0 is affected by ethnicity [9]. The maintenance doses of warfarin for the Japanese and the Chinese are about 30% and 40% lower than those of Caucasians, respectively [10]. Actually, genetic determinants of warfarin dosing may affect the effect of warfarin [11, 12]. Several studies evaluated GIB complications associated with warfarin in Western countries but the data of Asian population was rarely reported. This study investigated the incidence, characteristics, risk factors, management, and outcome of GIB in Taiwanese patients treated with warfarin anticoagulation therapy.

## 2. Materials and Methods

### 2.1. Study Design

This was a retrospective study of a cohort of warfarin anticoagulated patients in Kaohsiung Veterans General Hospital. We searched the electronic medical records of all patients with prescriptions of warfarin between July 1993 and May 2012. Patients were enrolled if they met the following criteria: (1) age equal to or more than 20 years old and (2) taking warfarin for more than 6 weeks. We reviewed the medical records and retrieved the information including age, gender, the indications and duration of warfarin therapy, concomitant medication during anticoagulation such as antiplatelet agents, nonsteroid anti-inflammatory drugs, and steroids, comorbidity, and INR values during anticoagulation. We further divided all patients into GIB group and non-GIB group. For patients with GIB, the symptoms and signs of GIB at presentation, needs of transfusion, endoscopic findings and therapies, duration of discontinuation of warfarin, and outcome of the patients were recorded. This study was approved by the Institutional Review Board of Kaohsiung Veterans General Hospital (VGHKS12-CT11-01).

### 2.2. Definition

The average INR value was determined as the mean of all INR values measured during anticoagulation and before GIB, if present. GIB was defined as (1) clinically evident hematemesis, melena, and hematochezia, or positive for occult blood test, and (2) needs of transfusion of two or more units of packed red blood cells, or a decline in hemoglobin level of 2 g/dL or greater, or a systolic blood pressure < 100 mmHg in patients negative for evident signs of GIB or occult blood test.

### 2.3. Statistical Analysis

The primary aim of this study was the incidence of GIB in patients treated with warfarin. The secondary aims were the risk factors of GIB and the outcome of the patients with GIB. The incidence of GIB was calculated as values per patient-year in this cohort. Demographic data were compared between patients of GIB group and non-GIB group. Categorical data were compared using chi-square or Fisher's exact tests as appropriate. Continuous variables with normal distributions were compared using independent Student's* t*-test. Continuous variables without normal distributions were compared using Mann-Whitney* U* test. A receiver operating characteristic (ROC) curve was used to determine the cut-off value of the mean INR which best discriminated GIB patients from non-GIB patients. Risk factors of GIB were examined by univariate and multivariate Cox regression analysis. Significance was defined as *P* < 0.05 for all two-tailed tests. All analyses were conducted by using SPSS software (version 12; SPSS Inc., Chicago, IL).

## 3. Results

A total of 401 warfarin anticoagulated patients were enrolled into this study. There were 234 males and 167 females. The mean age of the patients was 65.2 ± 16.6 years. The indications of warfarin were as follows: valvular replacement in 148 patients (36.9%), atrial fibrillation in 89 patients (22.1%), deep vein thrombosis in 71 patients (17.7%), pulmonary embolism in 31 patients (7.7%), and other conditions in 62 patients (15.4%). There were 36 patients in GIB group and 365 patients in non-GIB group. Demographic data of both groups were shown in Table 1. Patients with GIB were older (*P* < 0.001) and had a higher mean INR value (*P* = 0.01) than patients without GIB. Besides, the proportion of a history of GIB (*P* = 0.003), concomitant cirrhosis (*P* < 0.001), and septic shock (*P* < 0.001) was also significantly higher in GIB group patients than in non-GIB group patients.

### 3.1. Incidence and Characteristics of GIB

Thirty-six patients had at least one episode of GIB. The incidence of GIB was 3.9% per patient-year. There were totally 43 bleeding episodes in this cohort and the average episode of GIB was 1.1 per patient. The time between the use of warfarin and the first onset of GIB was 41.0 ± 58.4 months. Twelve patients (36.0%) had the first episode of bleeding within the first month after the start of warfarin anticoagulation. The presenting symptoms and signs of GIB included melena (15 patients, 41.7%), hematemesis (9 patients, 25.0%), hematemesis with melena (6 patients, 16.7%), hematochezia (3 patients, 8.3%), and a decrease of hemoglobin level more than 2 g/dL but negative for evident signs of GIB or occult blood test (3 patients, 8.3%).

Twenty-five of the 36 GIB patients underwent esophagogastroduodenoscopy (EGD) or colonoscopy according to the presentation of GIB. The causes of GIB included esophageal ulcer (1 patient, 4.0%), gastric ulcer (1 patient, 4.0%), duodenal ulcer (16 patients, 64.0%), gastric polyp (1 patient, 4.0%), rectal cancer (2 patients, 8.0%), and pseudomembranous colitis (1 patient, 4.0%) (Table 2). However, no identifiable source of bleeding could be found in 3 patients (12.0%). One of these patients received both EGD and colonoscopy and the other two patients underwent EGD only because they refused colonoscopy. No enteroscopy or angiography was performed in these patients.

### 3.2. Risk Factors of GIB

We further stratified the patients according to the mean INR values and found that the incidence of GIB increased with higher intensity of anticoagulation (Table 3). A ROC curve found that a mean INR of 2.1 was the cut-off value which best discriminated patients with GIB from patients without GIB. Patients with advanced age also had a trend towards a higher incidence of GIB, especially in patients aged more than 70 years old (Table 4).

Univariate analysis with Cox regression showed that GIB was significantly related to age > 65 years old (relative risk (RR): 2.5, 95% confidence interval (CI): 1.3–5.3, *P* = 0.02), a mean INR value > 2.1 (RR: 2.4, 95% CI: 1.2–4.5, *P* = 0.01), a history of GIB (RR: 4.6, 95% CI: 1.8–11.9, *P* = 0.002), and cirrhosis (RR: 8.9, 95% CI: 2.7–29.9, *P* < 0.001) (Table 5). Multivariate analysis with Cox regression revealed that age >65 years old (RR: 2.5, 95% CI: 1.2–5.5; *P* = 0.02), a mean INR > 2.1 (RR: 2.1, 95% CI: 1.0–4.2; *P* = 0.04), a history of GIB (RR: 5.1, 95% CI: 1.9–13.5, *P* = 0.001), and cirrhosis (RR: 6.9, 95% CI: 2.0–24.5, *P* = 0.003) were independent factors predicting GIB after adjustment (Table 6).

### 3.3. Management of GIB and Outcome

Intravenous vitamin K was administered in all GIB patients. Patients were transfused with 2.8 ± 5.0 units of packed red cells and 2.7 ± 6.3 units of fresh frozen plasma. Eight patients underwent endoscopic treatments including hemoclipping (4 patients), endoscopic injection therapy (3 patients), and argon plasma coagulation (1 patient). Uncontrolled bleeding was noticed in 2 patients with a history of GIB and cirrhosis. Warfarin was restarted in 22 patients (61.1%) in 7.9 ± 6.5 days after the GIB was controlled and none of these patients had thromboembolic events. However, recurrent GIB was found in 6 patients (27.3%). Of these patients, 1 patient presented with recurrent gastric ulcer bleeding, 2 patients presented with recurrent duodenal ulcer bleeding, and 2 patients presented with gastric cancer bleeding. One patient was among the patients with a decrease of hemoglobin level of more than 2 g/dL but negative for evident signs of GIB or occult blood test at the first bleeding episode and no bleeder could be identified at the rebleeding episode either. Warfarin was not restarted in 12 patients and none of them had recurrent GIB while cerebrovascular accident was noticed in 2 patients (16.7%). Fourteen GIB patients died. The causes of mortality included sepsis (6 patients), pneumonia (2 patients), subdural hemorrhage (1 patient), heart failure (2 patients), acute myocardial infarction (1 patient), and uncontrolled GIB (2 patients). In all 401 patients, the incidence of cerebral vascular accident and myocardial infarction was 1.2% (5 patients) and 0.7% (3 patients), respectively.

## 4. Discussion

We investigated the incidence, risk factors, management, and outcome of GIB in a cohort of warfarin anticoagulated Taiwanese patients. GIB occurred at an incidence of 3.9% per patient-year in this study. Age > 65 years old, a mean INR value > 2.1, a history of GIB, and cirrhosis were found to be independent risk factors of GIB. Warfarin was restarted in 61.1% of the GIB patients and 27.3% of them had recurrent GIB. Thromboembolic events were found in 16.7% of the patients who discontinued treatment of warfarin because of GIB.

GIB occurs at a rate ranging from 0% to 67% with an average bleeding rate of 3% in patients treated with warfarin [5]. The incidence of life-threatening and fatal hemorrhage episodes is around 5% and 1%, respectively [5]. Among our GIB patients, up to 33.3% of them had the first bleeding episodes within the first month of anticoagulation and 61.1% of the GIB occurred within the first year, which was consistent with the results of a meta-analysis [5]. This might be due to unstable intensity of anticoagulation during the early dosage adjustment period. As the INR values reach a stable therapeutic range, the bleeding incidence might decrease thereafter.

Polymorphisms in VKORC1 and CYP2C9 genes are associated with reduced doses of warfarin [12, 13]. The effect of warfarin is affected by genetic determinants of warfarin dosing, which may vary between different races. In our study, the bleeding incidence was similar to those of Western studies. It is possible that the genetic polymorphisms are similar between different races so the dosing of warfarin may only play a partial role in addition to anticoagulation intensity, drug interactions, and underlying diseases. Recently, two large randomized controlled trials compared clinically guided dosing with genotype-guided dosing of warfarin in patients initiating anticoagulant therapy, and genotype-guided dosing strategy did not result in a better outcome in both studies [14, 15]. Therefore, close monitoring of INR testing is still the most important issue in patients initiating warfarin anticoagulation.

Several risk factors were found to be associated with GIB in this study. The incidence of GIB increased with the increment of the mean INR values, and a mean INR of 2.1 best discriminated the patients with and without GIB and patients with a mean INR of 3.0 or more carried the highest risk of GIB. This was not surprising because intensity of anticoagulation highly correlates with the risk of bleeding in warfarin anticoagulated patients in most studies [4, 5, 16]. Age > 65 years old was found to be significantly associated with GIB. Slow metabolized rate of warfarin, an elevated risk of drug interactions because of polypharmacy, and chronic illness were proposed to increase the risk of bleeding in elderly patients [17]. Whether older age increases the risk of bleeding in patients treated with warfarin is controversial [5]. However, it is well known that persons older than 80 or 85 years of age do carry a significant risk of bleeding [17, 18]. Therefore, warfarin should be cautiously used in elderly patients, especially in extremely old patients. Chronic liver disease had been shown to increase bleeding risks in hospitalized patients [19]. Although the mechanism is unclear, the relative deficiency in vitamin K dependent clotting factors causing bleeding tendency as seen in cirrhotic patients may play a role. We also found that a history of GIB increased the risk of GIB. Actually, concurrent use of proton pump inhibitors significantly reduces risk of GIB in patients treated with warfarin [20]. Therefore, acid suppressants may be considered in patients with a history of GIB during warfarin anticoagulation.

Most of the GIB events were controlled by vitamin K administration, blood transfusion, acid suppression, or endoscopic therapies. However, two fatal GIB events were noticed within the first month of warfarin anticoagulation. Both patients had a history of GIB and underlying liver cirrhosis. This suggested that patients with multiple risk factors of GIB might need more frequent testing of INR values, especially in the early stage of warfarin anticoagulation.

The pros and cons of restarting warfarin in the patients with GIB are rarely evaluated. It was noteworthy that warfarin was restarted in only 61.1% of our GIB patients and 27.3% of them experienced recurrent GIB while thromboembolic events were noticed in 16.7% of the patients who did not continue warfarin therapy. The chance of rebleeding after successful hemostasis depends on the cause of bleeding [4], and patients with independent risk factors for GIB should be thoroughly evaluated before restarting warfarin. Long-term acid suppressants may be considered in patients with peptic ulcer bleeding. Besides, a policy of lower-intensity anticoagulation may be beneficial in most of the cases. A warfarin dose adjusted to maintain an INR of 1.4 or more was found to be effective in the primary prevention of coronary heart disease [21]. Recent guidelines also recommended using lower-intensity anticoagulation in patients older than 75 years of age [22]. To prevent the thromboembolic event while minimizing the risk of GIB, an anticoagulation intensity at an INR of 2.0 or less could be an alternative anticoagulation strategy to traditional range, that is, 2.0 to 3.0.

Some limitations do exist in this study. First, it was a retrospective study and the GIB rate may be underestimated because some GIB patients might not present to this hospital. Second, the pharmacogenetics information such as polymorphisms in VKORC1 and CYP2C9 genes was not available in this study. Third, only 70% of our GIB patients underwent endoscopy, which was similar to a previous study [3]. Besides, the bleeding source was not identified in 12% of the patients undergoing endoscopic examination. Excessive anticoagulation intensity may contribute to the low diagnostic rate of endoscopy [23]. Fourth, some patients were missing the information of the status of* Helicobacter pylori* (*H. pylori*) infection. Patients with* H. pylori* infection are well known to be at a higher risk of GIB. Whether eradication of* H. pylori *can decrease the risk of GIB in warfarin anticoagulated patients needs to be studied in the future. Finally, although cirrhosis and a history of GIB were found to be independent risk factors for GIB, the possibility of overinterpretation could not be overlooked because the numbers of these patients were relatively small and the confidence intervals were wide during subgroup analysis.

## 5. Conclusions

Our study found that warfarin therapy carried a similar risk of GIB in Taiwanese patients as compared with most Western studies. Old age, intensity of anticoagulation, a history of GIB, and advanced liver disease were associated with GIB. To decrease the risk of GIB while maintaining the effect of anticoagulation, frequent testing of INR values and a strategy of low to moderate intensity of anticoagulation could be considered in patients with risk factors of GIB. In terms of restarting warfarin following hemostasis in patients with GIB, long-term acid suppressants could be used in addition to more close monitoring of INR values and low to moderate intensity of anticoagulation.

## Figures and Tables

**Table 1:** Demographic data of patients treated with warfarin.

Variables	Patients without GIB* (*n* = 365)	Patients with GIB* (*n* = 36)	*P* value
Age (year)	64.1 ± 17.0	74.3 ± 12.3	<0.001
Male	215	19	0.5
Smoking	101 (27.6%)	10 (27.7)	1.0
Alcohol	73 (20.0%)	6 (16.6%)	0.6
Indications for warfarin			
Deep vein thrombosis	63 (17.2%)	8 (22.2%)	0.5
Pulmonary embolism	28 (7.6%)	3 (8.3%)	0.9
Valvular replacement	138 (37.8%)	10 (27.7%)	0.2
Atrial fibrillation	81 (22.1%)	8 (22.2%)	1.00
Others	55 (15.0%)	7 (19.4%)	0.5
Platelet count (K/cumm)	201.6 ± 139.2	206.8 ± 144.5	0.9
Mean INR* value	1.8 ± 1.2	2.2 ± 1.7	0.01
History of GIB^†^	12 (3.2%)	5 (13.8%)	0.003
Comorbidity			
Hypertension	200 (54.8%)	22 (61.1%)	0.5
Cardiovascular disease	62 (16.9%)	6 (16.6%)	1.0
Pulmonary disease	32 (8.7%)	6 (16.6%)	0.1
Cirrhosis	1 (0.2%)	3 (8.3%)	<0.001
Renal insufficiency	27 (7.3%)	4 (11.1%)	0.4
Malignancy	38 (10.4%)	5 (13.9%)	0.6
Septic shock	2 (0.5%)	3 (8.3%)	<0.001
Concomitant medication			
NSAID^‡^	6 (1.6%)	2 (5.5%)	0.1
Aspirin	46 (12.6%)	4 (11.1%)	0.8
Clopidogrel	30 (8.2%)	1 (2.7%)	0.2
Dipyridamole	22 (6.0%)	5 (13.8%)	0.07
Steroids	25 (6.8%)	4 (11.1%)	0.3

*INR: international normalization ratio.

^†^GIB: gastrointestinal bleeding.

^‡^NSAID: nonsteroidal anti-inflammatory drug.

**Table 2:** Endoscopic findings of warfarin anticoagulated patients with gastrointestinal bleeding.

Source of bleeding	Patients (%)
Upper gastrointestinal bleeding	
Esophageal ulcer	1 (4.0%)
Gastric ulcer	1 (4.0%)
Duodenal ulcer	16 (64.0%)
Gastric polyp	1 (4.0%)
Lower gastrointestinal bleeding	
Rectal cancer	2 (8.0%)
Pseudomembranous colitis	1 (4.0%)
No identifiable source	3 (12.0%)

**Table 3:** Incidence of gastrointestinal bleeding in relation to international normalization ratio.

INR*	Events	Patients	Follow-up (months)	Events/patient-years
≤1.0	0	0	112	0
1.0–1.5	6	5	3278	2.1
1.5–2.0	18	14	5412.5	3.9
2.1–2.5	11	9	2874	4.5
2.5–3.0	2	2	887	4.5
>3.0	6	6	503.1	14.3

*INR: international normalization ratio.

**Table 4:** Incidence of gastrointestinal bleeding in relation to age.

Age	Events	Patients	Follow-up (months)	Events/patient-years
≤40	0	0	869	0
41–50	2	2	1273	1.8
51–60	6	5	2336	3.0
61–70	5	4	2115.5	2.8
71–80	13	10	3667	4.2
>80	17	15	2806.1	7.2

**Table 5:** Univariate analysis of risk factors of gastrointestinal bleeding in patients taking warfarin.

Variable	Relative risk	95% confidence interval	*P* value
Age > 65 yrs	2.5	1.3–5.3	0.02
Mean INR* > 2.1	2.4	1.2–4.5	0.01
History of GIB^†^	4.6	1.8–11.9	0.002
Cirrhosis	8.9	2.7–29.9	<0.001
Gender	0.6	0.3–1.2	0.2
Smoking	1.0	0.5–2.3	0.8
Alcohol	0.7	0.3–61.8	0.5
Hypertension	0.6	0.8–3.3	0.1
Cardiovascular disease	1.5	0.7–3.1	0.2
Pulmonary disease	1.3	0.5–3.2	0.5
Renal insufficiency	1.1	0.4–3.2	0.8
Malignancy	1.2	0.5–3.0	0.3
Septic shock	3.0	0.9–10.1	0.08
NSAID^‡^	3.0	0.7–12.7	0.1
Aspirin	0.9	0.3–2.7	1.0
Clopidogrel	0.5	0.07–3.9	0.5
Dipyridamole	2.4	0.9–6.2	0.08
Steroids	1.6	0.5–4.6	0.4

*INR: international normalization ratio.

^†^GIB: gastrointestinal bleeding.

^‡^NSAID: nonsteroidal anti-inflammatory drug.

**Table 6:** Multivariate analysis of risk factors of gastrointestinal bleeding in patients taking warfarin.

Variable	Relative risk	95% confidence interval	*P* value
Age > 65 yrs	2.5	1.2–5.5	0.02
Mean INR* > 2.1	2.1	1.0–4.2	0.04
History of GIB^†^	5.1	1.9–13.5	0.001
Cirrhosis	6.9	2.0–24.5	0.003

*INR: international normalization ratio.

^†^GIB: gastrointestinal bleeding.
